# *Cryptococcus gattii*: Emergence in Western North America: Exploitation of a Novel Ecological Niche

**DOI:** 10.1155/2009/176532

**Published:** 2009-01-15

**Authors:** Kausik Datta, Karen H. Bartlett, Kieren A. Marr

**Affiliations:** ^1^School of Medicine, Johns Hopkins University, 720 Rutland Avenue, Room 1064, Ross Building, Baltimore, MD 21205, USA; ^2^School of Environmental Health, University of British Columbia, Vancouver, BC, Canada V6T 1Z3

## Abstract

The relatively uncommon fungal pathogen *Cryptococcus gattii* recently emerged as a significant cause of cryptococcal disease in human and animals in the Pacific Northwest of North America. Although genetic studies indicated its possible presence in the Pacific Northwest for more than 30 years, *C. gattii* as an etiological agent was largely unknown in this region prior to 1999. The recent emergence may have been encouraged by changing conditions of climate or land use and/or host susceptibility, and predictive ecological niche modeling indicates a potentially wider spread. *C. gattii* can survive wide climatic variations and colonize the environment in tropical, subtropical, temperate, and dry climates. Long-term climate changes, such as the significantly elevated global temperature in the last 100 years, influence patterns of disease among plants and animals and create niche microclimates habitable by emerging pathogens. *C. gattii* may have exploited such a hitherto unrecognized but clement environment in the Pacific Northwest to provide a wider exposure and risk of infection to human and animal populations.

## 1. Introduction: The Organism and the Disease

*Cryptococcus gattii* (formerly, *Cryptococcus neoformans* var. *gattii*) [1] is a basidiomycetous yeast pathogenic to immunocompetent mammals including humans. This relatively uncommon organism differs from the congeneric, more commonly encountered pathogen, *C. neoformans*, with regards to phenotypic characteristics, natural habitat, epidemiology, ecology, clinical manifestations of disease, and responses to antifungal therapy [2]. Phylogenetic studies have shown that *C. gattii* and *C. neoformans* diverged from a common ancestor approximately 40 million years ago [3]. Hosts acquire cryptococci via inhalation, and the disease (“Cryptococcosis”) caused by both *C. gattii* and *C. neoformans* affects the lungs, with the potential to disseminate to distant tissues, most frequently, the central nervous system, causing life-threatening meningoencephalitis [4]. Compared to *C. neoformans*, *C. gattii* infections more often cause granulomatous lesions (cryptococcomas) in lung and brain, with more associated neurological sequelae and morbidity [5–8]. Though otherwise healthy hosts presenting with meningitis respond to antifungal therapy, complete mycological cure (culture negativity) appears to be more often delayed [7, 9]. Genetic typing studies using molecular techniques have identified distinct haploid genotypes among the clinical, veterinary, and environmental isolates of *C. gattii*, namely, VGI, VGII (further subdivided into VGIIa, VGIIb [10, 11], and recently VGIIc [12]), VGIII, and VGIV [11–15]. The genotype VGIIa was preponderantly present in the emergence of *C. gattii* disease in 1999 on Vancouver Island and Lower Mainland of British Columbia (BC) in Canada, and has, therefore, been termed the “Vancouver Island major emergence strain”; it was found to be unique to the Pacific Northwest and hypervirulent in mouse studies [10, 11].

## 2. *C. gattii* Outbreak in the Pacific Northwest

*C. gattii* emerged as an agent of life-threatening infections in the Pacific Northwest of North America in 1999. Previously unknown in the region, more than 200 cases of *C. gattii* have now been documented in humans, and more than twice that number in domestic animals, accounting for an average annual incidence of 6.5 cases per million in BC or 27.9 cases per million on Vancouver Island; this is the highest “endemic” incidence reported worldwide [16]. There has been a substantial mortality from this disease; between 1999 and 2006, the case fatality rate from *C. gattii* disease was estimated to be 4.5% [17].

Although the majority of locally acquired *C. gattii* cases occurred in Vancouver Island residents, since 2004 there has been a steady increase in the numbers among BC Lower Mainland residents [17] as well as among the Northwestern states in the United States (Washington and Oregon) [10, 11, 16, 18–20]. To date, we are aware of approximately 20 cases of *C. gattii* disease diagnosed in humans in Washington and Oregon, many of whom have had no travel history to any known *C. gattii*-disease endemic zone (K Marr, unpublished observation). The organism has also been recovered from the environment in Washington State [16]. An overwhelming majority of these isolates belonged to the molecular genotype VGII (∼95% VGIIa; ∼5% VGIIb) and one VGIII [10–12, 14, 16, 19, 20]. Though the exact mode of transport of *C. gattii* from Vancouver Island to the Lower Mainland is not known, studies of possible dispersal mechanisms have indicated association of *C. gattii* cases with high-traffic locations, and evidence of human-associated dispersal through wheel wells of cars, shoes, and movement of soil or wood products as well as water [18, 21].

Since 1999, the incidence of *C. gattii* infection in domestic animals has increased greatly in Western North America, in parallel with the outbreak of human cryptococcosis. *C. gattii* disease has been diagnosed in dogs, cats, ferrets, porpoises, alpacas and llamas, horses, and psittacine birds on Vancouver Island, BC Lower Mainland [22–24] as well as in Washington and Oregon [12, 16, 20]. Cryptococcal infection in animals occurs from inhalation of airborne infectious propagules (yeast or spores) and subsequent colonization of the nasal cavity and paranasal sinuses, often resulting in asymptomatic carriage and/or subclinical infection [2, 22]. Subclinically infected animals may clear the organism, remain protractedly colonized and/or infected (with repeatedly positive serum for cryptococcal antigen), or progress to clinical disease [25]. Disease can be manifested by upper respiratory symptoms, subcutaneous nodules, pneumonia, central nervous system and/or ocular disorders, and lymphadenopathy [2, 24]. A unique feature of the Pacific Northwest outbreak is the number of marine mammals, primarily porpoises, which have died with *C. gattii* pneumonia [24]. Over 90% of the veterinary isolates have belonged to molecular type VGIIa [16, 22–24]. Identified risk factors in animals in this region include disturbance of soil and/or human activities such as logging within 10 km, hiking, or hunting in areas colonized with *C. gattii* [26].

## 3. *C. gattii*: Establishment in a Novel Environmental Niche

In a seminal study on the epidemiology of cryptococcosis [27], it was noted that the majority of clinical cases of *C. gattii* disease occurred in people residing in hot and humid climates of the tropics and subtropics. Prior to 1999, the geographical regions with any degree of *C. gattii* endemicity included Australia and New Zealand, Papua New Guinea, South and Southeast Asia (Cambodia, Malaysia, Thailand, Vietnam, China, Taiwan, Singapore, Nepal, and the Indian subcontinent), parts of Latin America (Argentina, Brazil, Colombia, Uruguay, Paraguay, Peru, and Venezuela), Southern California, Mexico, Hawaii, Central and South Africa, and certain parts of Europe (Austria, Germany, France, Italy, Greece, and Spain) (reviewed in [2]; also see [28–32]).

Studies from Australia and elsewhere [2] indicate that *C. gattii* can colonize the environment in tropical, subtropical, temperate, and dry climates. Although *C. gattii* grows slower at 37°C compared to *C. neoformans* isolates [33], environmental isolates of *C. gattii* grow equally well at 30°C and 37°C—a survival property believed to be mediated in part by a manganese-containing mitochondrial superoxide dismutase (*SOD2*) which is induced at elevated temperatures [34]. Recent work with environmental isolates from Vancouver Island has shown that the organism, when grown in nutritionally deficient soil extract broth, uses constituents in the soil to produce melanin; these melanized cells are more resistant to UV irradiation exposure than the same strains which did not melanize when grown in 1/16th strength malt extract. The ability of this *C. gattii* genotype to resist UV irradiation and colonize with high concentrations in desiccated soil may contribute to the survival and dispersal of this strain in this environment [35].

The origins of the *C. gattii* strains, which now permanently colonize the environment on Vancouver Island, remain unknown. The provincial medical mycology collection has been examined, and no archived *C. gattii* isolates were identified prior to 1999. Significant global dispersal of cryptococcal strains [14, 36] makes accurate determination of specific origin difficult. However, genotypic analysis enabled the study of lineages of the *C. gattii* isolates, tracing the origin and evolution of the organism and establishing its relevance in the context of the outbreak. All *C. gattii* clinical isolates from the Vancouver Island emergence were found to contain *α* allele of the mating type-specific genes (MAT*α*) and were sexually fertile [11, 37]. Genotypic analysis further revealed that a rare, nonclassical, same-sex reproduction between two MAT*α* parents resulted in the VGIIa strain. These observations support the hypothesis that cryptic same-sex reproduction may have enhanced the virulence of the VGIIa genotype, helping a “hypervirulent” strain adapt to and propagate in the local environmental niche [10]. In contrast, study of Australian *C. gattii* populations recovered from Eucalyptus tree hollows found that the organism exists as either *α*-mating-type isolates, or both a and *α*-mating-type isolates, and both unisexual and heterosexual recombination produce infectious spores [38].

Because of global dispersal [14, 36], it is possible that the Vancouver Island major genotype VGIIa may exist elsewhere, but that remains to be established; one South American isolate thus far reported to be highly similar to VGIIa differs at one locus by multilocus sequence typing (MLST). It may well be related to the major outbreak strain, but either with a different origin (in Australia, South America, or within the Pacific Northwest), or by genetic drift (in transit to the Pacific Northwest, perhaps by mating with another VGII strain). On the other hand, the minor genotype VGIIb appears to be identical with fertile isolates from Australia and likely originated there [10].

In the first two Oregon cases of nontravel-associated *C. gattii* disease, *C. gattii* isolates genotyped as VGII, but they were genetically distinct from either VGIIa or VGIIb strains found on Vancouver Island [16] and the relationship of these cases to the outbreak is unknown. However, two archived *C. gattii* isolates from the United States, NIH444 (a.k.a. ATCC 32609 and CBS6956) and NIH B4534 (a.k.a. CBS7750), were found to be identical to the Vancouver Island VGIIa strain [10, 11], leading to the hypothesis that this genotype may have been present in North America for more than 30 years. Strain NIH444 was isolated from a sputum sample from a patient in Seattle, Washington, in the early 1970s, and strain NIH B4534 was recovered from a Eucalyptus tree in San Francisco in 1992. It is important to note that we do not have a complete travel history from the Seattle patient. However, if this isolate has been in the environment in Western North America for this long, other factors, such as changing conditions of climate or land use and/or host susceptibility, have appeared to encourage its emergence in more recent years.

## 4. Climate Change and Disease Relationship: Overview

In the last 100 years (1906–2005), the global temperature has increased by 0.74 [CI, 0.56–0.92]°C; and the linear warming trend over the last 50 years (0.13 [CI, 0.10–0.16]°C per decade) is nearly twice that for the last 100 years [39]. The last three decades have seen an unprecedented escalation of global warming—both at the level of the middle troposphere and the surface—a large part of it being anthropogenic through emission of greenhouse gases. Long-term changes in climate and subsequent effects have been observed at continental, regional, and ocean basin scales, including changes in Arctic temperatures and ice, widespread changes in humidity and precipitation amounts, ocean level and salinity, wind patterns, and aspects of extreme weather including droughts, heavy precipitation, heat waves, and the intensity of tropical cyclones. It has been predicted that the earth in the 21st century will face some 2°C (3.5°F) or more in additional warming [39]. However, the rate of change between microenvironments varies substantially, with polar zones changing more quickly while other zones remain relatively stable, and therefore, global averages cannot be used to predict effects of microclimates. Since 1948, the average annual temperatures at one site on the eastern coast of Vancouver Island increased by 1.44°C which is a statistically significant change (*P* < .001) (data obtained from Environment Canada). Unfortunately, many government sponsored meteorological stations have been abandoned, leaving research scientists with the arduous task of documenting microclimate variation.

Speculative studies on how a widespread climate change, specifically the elevation of global temperature, might affect the distribution of infectious diseases began about two decades ago. Health outcomes of climate change are diverse, and depend upon many different factors with respect to biodiversity, nutrient cycle, physical relocation, internal defense systems, and transmission dynamics within microbes [40]. The initial studies almost exclusively focused on vector-borne diseases, predicting possible vector movements with rise in temperature in erstwhile temperate zones. However, the relationship between climate change and infectious disease is inherently complex, and not easily amenable to predictive epidemiology. For example, the impact of climate change on infectious disease is not restricted to vector-borne diseases or infections that affect human health directly. Climate change may influence patterns of disease among plants and animals, impacting the human food supply (and thereby reducing human resistance to infections), or indirectly causing human disease patterns to shift, as the host range for disease reservoirs may change because of human migration to geographically disparate areas and/or changes in abundance and distribution of disease vectors and agents [40–42].

In 2004, the World Health Organization (WHO) published a study of the global disease burden attributable to human-induced climate change up to the year 2000, and made quantitative model-based predictions of climate change-associated health risks up to 2030 [43]. Despite the lack of comprehensive models for various specific climate-disease relationships, overall results indicate that even the subtle climatic changes occurring since the mid-1970s could be responsible for over 150 000 deaths from climate-sensitive diseases, and approximately 5 million disability-adjusted life years each year—the most vulnerable being the poorer regions of the world, according to WHO estimates, although climate change poses a global threat to public health [43]. Diseases with which significant association of climate changes has already been observed include water- and food-borne diseases (such as cryptosporidial diarrhea, salmonellosis, algal toxicity, and cholera), vector-borne diseases (such as malaria, Chagas disease, borreliosis, schistosomiasis, and dengue), rodent-borne diseases (such as leptospirosis) as well as infections caused by the St. Louis Encephalitis virus and the West Nile virus, both of which have been shown to prefer warmer climates [42]. The global warming phenomenon has, in addition, been associated with various other noninfectious diseases with significant human morbidity, including chronic respiratory ailments, cardiovascular diseases, neurological and psychiatric disorders as well as those pertaining to occupational health [44]. According to the WHO estimates of morbidity and mortality associated with anthropogenic climate change, the year 2030 would see a doubling of excess risk of the various health outcomes [43].

## 5. *C. gattii*: Change of Environmental Niche or Adaptation to a New One

In Australia, in the early 1990s, Ellis and Pfeiffer observed a correlation between the distributions of human cryptococcal disease and of Eucalyptus trees [45, 46], whose bark is rich in dihydroxyphenylalanine (L-DOPA), a substance metabolized by *Cryptococcus* species to produce melanin; this has been hypothesized to contribute to their environmental survival [47, 48]. Association of *C. gattii* with Eucalyptus has been sporadically reported from India, Brazil, Italy, and the United States [49–52]. However, the absence of this association with Eucalyptus in other endemic areas, such as the Northern Territory of Australia [53], Papua New Guinea [54], Central and South Africa [55], Brazil, and Malaysia as well as the isolation of *C. gattii* from non-Eucalyptus trees and tree materials [56–60], indicates the existence of additional environmental sources. Since it was first discovered to have a stable ecological niche on Vancouver Island in 2002, *C. gattii* has consistently been recovered in high concentration from native trees, soil, air, freshwater, and seawater [16, 19].

Does the Vancouver Island outbreak, then, point to a changing ecological niche for this organism in Western North America? From the beginning of the *C. gattii* epidemic on Vancouver Island and adjoining areas, epidemiological studies showed that all humans and animals with cryptococcal infection either lived within or traveled to areas identified by unique weather and vegetation zones. The British Columbia Ministry of Forests categorizes areas with similar ecologies within the province by biogeoclimatic zone designations. A biogeoclimatic zone is a geographical area with an ecosystem comprised of a relatively uniform macroclimate, defined vegetation, soils, and animal life inhabiting that climate, and may contain smaller ecosystems (subzones) that reflect differences in regional climate, soil moisture, soil nutrient status, and environmental disturbance [61]. The unique zones along the eastern edge of Vancouver Island are in the rain shadow of the Olympic Mountains located in Washington State to the south, and include the Coastal Douglas Fir (CDF) and very dry Coastal Western Hemlock (CWH) biogeoclimatic zones. The CDF and very dry CWH zones are characterized by warm, dry summers (average summer temperature 15.6 ± 1.24°C; 190.24 ± 55.5 mm rain), and mild, wet winters (average winter temperature 5.77 ± 0.64°C; 884.33 ± 206.22 mm rain). *C. gattii* has been repeatedly and consistently isolated from the environment in these biogeoclimatic zones [16, 19, 22]. The CDF and CWH zones also include the Southern Gulf Islands and portions of the BC Lower Mainland. Similar climates with comparable temperature and rainfall extend further south into Washington and Oregon in the United States. The San Juan Islands, Puget Sound in Washington, and the Willamette Valley in Oregon harbor ecologically similar plant diversity as in BC, lending support to the idea that environmental niches suitable for colonization by *C. gattii,* are present in Western North America [16]. Indeed, large-scale environmental sampling performed during 2001–2005 on the BC mainland, the Gulf Islands, and Washington revealed that 3% of 2 033 off-Vancouver Island samples of air, water, soil, and trees were positive for *C. gattii* serotype B (mostly VGIIa, except two VGIs) [16].

Environmental sampling studies on Vancouver Island revealed high concentrations of *C. gattii* in the soil, indicating a potential source of exposure [18], and data gathered from the BC environment conclusively demonstrate that *C. gattii* is well adapted for survival in a dry, nutrient-deprived soil and is more likely to spread as airborne propagules during dry summer weather [19]. However, whereas *C. gattii* was consistently isolated from localized areas, it was not found, or was below the limit of detection in other areas, such as the San Juan Islands [62], despite ecological similarity to identified zones of endemicity in Vancouver Island and BC Lower Mainland. This suggests that there may be environmental “hotspots”, *that is,* zones of high concentration, of *C. gattii* within the same broad ecological niches. There are also areas with transient colonization, with sites intermittently positive over time, and some sites which initially tested negative which subsequently either became colonized, or the concentration rose above the limit of detection [18, 19]. Whether microclimatic conditions govern the creation and maintenance of these hotspots is not known, but the fact remains that *C. gattii* now inhabits a stable ecologic niche in the environment of the Pacific Northwest in concentrations high enough to pose a risk of infection through environmental exposures. Prior to the outbreak of *C. gattii* disease, there was no reason to seek its presence in the environment, as there were no cases of locally acquired infection, and *C. gattii* is not a phytopathogen. The relationships between an emerging pathogen and the environment are not often studied, and it is even rarer that researchers have been able to document the emergence with as much detail as has been possible in this BC outbreak. The unexpectedly rich dataset obtained from BC environmental and epidemiological ecologies can be used to inform future eco-health investigations. In this case, the existence of libraries of cryptococcal strains from global clinical and environmental origins provided the resources for swift characterization and molecular epidemiology of the causative organism; this information changed forever the simplistic idea of unvarying geographic boundaries on infectious organisms.

A promising tool which can be used by climatologists working with epidemiologists to predict public health impacts is ecological niche modeling [63–65]. These computer simulations take into account ecosystems, meteorology, and the presence of pathogens in the environment to predict areas with similar ecologies that may be at risk of pathogen spread. A recently completed thesis from the University of British Columbia using the Generic Algorithm for Rule-set Prediction (GARP) modeled the potential for *C. gattii* to spread to other areas of the Pacific Northwest [66]. Using existing data from human and animal clinical cases and Geographic Information System (GIS) coordinates of colonized environmental sites [18, 19, 21, 24], the model predicts a larger area of potential colonization than is currently the case. The predicted area includes cities with large populations (over 4 million). Since this model was developed, there have been at least four-human, eight-animal, and four-environmental samples of *C. gattii* recovered from the predicted zone which was not on Vancouver Island [16, 17, 20]. By the same token, the vastly larger land mass of British Columbia is predicted not to become colonized with *C. gattii* in the foreseeable future due to the extremes of temperature, snow cover, or lack of suitable habitat [66].

*C. gattii*, similar to the congeneric pathogen *C. neoformans*, has evolved mechanisms which protect it from the environment. For example, the melanin production by these organisms contributes to the protection from the ultraviolet radiation in sunlight. The predominant genotype of the Vancouver Island outbreak thrives in dry, nutrient poor soil at high concentrations. It appears to be well adapted for this marginal microecologic zone [19]. In Colombia, for example, the dispersal of *C. gattii* is greater during periods of high humidity or rain [67]. In Australia, the airborne distribution of propagules is associated with the flowering season of Eucalyptus [68]. Neither of these conditions describes the experience of the British Colombian genotypes VGIIa or VGIIb, which suggests that the organism is successful in this new ecologic niche because it can adapt preexisting characteristics to fill novel, underutilized microscopic niches, rather than strictly requiring a constrained environment. Even the widely held view that *C. gattii* was restricted to a symbiotic relationship with Eucalyptus trees has since been shown not to be the case, as *C. gattii* regularly colonizes native trees in countries outside Australia [57, 58, 69].

Cyclical climate change patterns (called “oscillations”) are driven by forces such as solar cycles and the ocean currents characterized by the El Niño and La Niña years, whereas more long-term changes, such as an elevation of global temperatures, must be measured over decades or centuries. Therefore, the problem of association of climate changes with the epidemiology of various infectious diseases is twofold. Climate changes may be long term, with the potential to cause significant epidemiological changes over long-time horizons, or the climate may suddenly shift, changing patterns and spread of exposure in a given climatic zone. Both of these possibilities pose challenges to public health studies.

That *C. gattii* emerged as a human and animal pathogen in the late 1990s, in a new habitat, is indisputable. Whether or not the climatic conditions (both short- and long-term) in the new habitat made it conducive for the organism to do so is under debate. The oscillatory climate change patterns preceding the emergence were similar to the patterns seen in this area over the last 30 years. The years 1992–1994 and 1998–2003 were dryer than the 30-year average during the summer, followed by the years 1997, 2004, and 2005, which had higher-than-average summer rainfall; the winter rains roughly followed the same pattern. Importantly, however, the amount of snow coverage on southern Vancouver Island and the CDF biogeoclimatic zone decreased over the same time period. This constantly repeating dry/wet pattern in conjunction with the elevation of temperature may well be a driving factor in the prolongation of *C. gattii* as a pathogen in this geographic location. However, given the paucity of long-term data on *C. gattii* emergence in relation to climate changes, it is difficult to attribute the emergence entirely to climate change. A proof-of-concept example supporting the notion is *Coccidioides immitis*, an environmental organism which proliferates in wet years and is widely dispersed in dry years, causing localized spikes in hospitalization for areas adjacent to endemic locations [70]. For *C. gattii*, some factor(s) (climate, land use, human agency, etc.) must have become permissive to the permanent colonization of the organism in high enough concentration to cause disease with an incidence rate of around 28 cases/million population (on Vancouver Island alone) [16]. Identification of those factors would help formulate preventive public health approaches.

It is also clear that *C. gattii* disease has escalated to an “off island” problem as well, involving the Northwestern US (Washington and Oregon). The extent of disease and the distribution of the cryptococcal isolates within the United States are unknown, and will remain underappreciated until laboratory methods to identify and speciate the isolates, standardized reporting of disease, and environmental studies are established.

*C. gattii* already had the ability to survive in a wide range of environmental variations, but the Western North America outbreak teaches us that it may exploit hitherto unrecognized but clement environments and provide a wider exposure, and thereby, risk of infection to the human and animal populations. The challenge for public health is to coordinate efforts toward early recognition of the emergence of new or reemergence of previously encountered infectious diseases to alert primary health care providers as to the diagnosis and appropriate treatment in order to prevent excess morbidity and mortality. However, understanding the consequences of ecological change is a group effort which includes health researchers, climatologists, and ultimately global citizens whose health may depend on the capacity to adapt to a rapidly changing environment.
